# Pulmonary Embolism following Percutaneous Nephrolithotomy: An Uncommon and Life-Threatening Complication

**DOI:** 10.1155/2019/2186930

**Published:** 2019-01-31

**Authors:** Spyridon Paparidis, Antonios Katsimantas, Dimitrios Oikonomidis, Nikolaos Ferakis

**Affiliations:** ^1^Urology Department, Korgialenio-Benakio Hellenic Red Cross Hospital, Athanasaki 1, 11526 Athens, Greece; ^2^Second Cardiology Department, Korgialenio-Benakio Hellenic Red Cross Hospital, Athanasaki 1, 11526 Athens, Greece

## Abstract

High risk pulmonary embolism is a rare and life-threatening complication following percutaneous nephrolithotomy. We report the case of a previously healthy, 44-year-old male, who developed acute pulmonary embolism following right percutaneous nephrolithotomy. On the 1st postoperative day, the patient presented with hemodynamic instability, acute respiratory distress, hypoxia, and loss of consciousness. He was urgently intubated and placed on mechanical ventilation. Clinical findings set the suspicion of pulmonary embolism with shock. Chest computed tomography scan confirmed the diagnosis. The patient underwent urgent thrombolysis in the cardiac care unit. On the 2nd postoperative day, the patient was admitted to the intensive care unit due to hemodynamic instability and fever. The postoperative course was complicated by right renal bleeding on the 3rd postoperative day, which was managed through angiography and angioembolization of the lower segmental right renal artery, followed by recurrent respiratory and urinary tract infections. The patient was transferred back to the urology department on the 66th postoperative day and was discharged seven days later.

## 1. Introduction

Complications of percutaneous nephrolithotomy (PCNL), according to the literature, are minor ones such as extravasation (7,2%), renal hemorrhage (0,6-1,4%), blood transfusion (11,2-17,5%), and fever (21-32,1%), which can be managed conservatively or minimally invasively on early diagnosis, and major ones such as septicemia (0,3-4,7%), colonic injury (0,2-0,8%), and pleural injury (0-3,1%) [1]. Complication rates vary from 20 to 83% [2]. Death rates after PCNL range from 0,1 to 0,7% [2]. Pulmonary embolism (PE) and myocardial infraction occur in less than 3% of patients undergoing PCNL [2]. Our aim is to present an interesting and uncommon complication of PCNL.

## 2. Case Report

**Figure 1:**
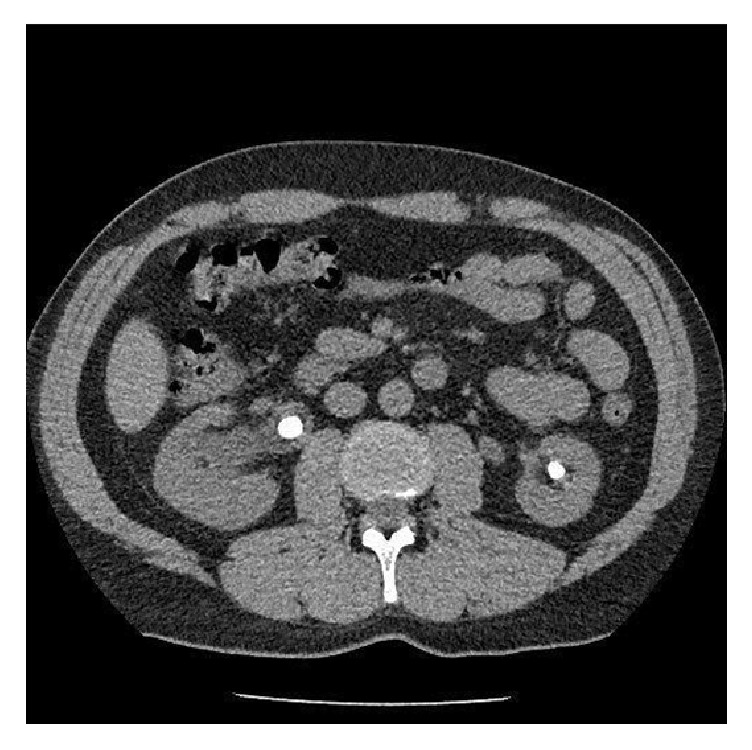
Preoperative CT scan. Bilateral nephrolithiasis.

A 44-year- old Caucasian male, with body mass index: 29.3, was admitted to our department due to bilateral nephrolithiasis, in order to undergo right PCNL. Preoperative computed tomography (CT) scan demonstrated a 22 mm calculus in the right renal pelvis, causing pyelocaliceal obstruction and dilatation, and smaller calculi in the lower calyceal group of the ipsilateral kidney. Multiple calculi in the upper, median, and lower calyceal group of the left kidney were also present, with no signs of pyelocaliceal obstruction (Figure 1). The patient had no medical history and the preoperative urine culture was negative.

The patient underwent right PCNL in prone position under general anesthesia. Following placement of a 7Fr ureteral catheter in lithotomy position and injection of contrast agent, a single tract at the median calyceal group was created above the 12th rib under fluoroscopy. After tract dilation with a balloon dilator, a 30Fr Amplatz sheath was positioned inside the calyx of puncture. Following stone fragmentation with a pneumatic lithotripter, the stones were removed using grasping forceps and an 18Fr nephrostomy tube was inserted for postoperative drainage. There were no intraoperative complications and the patient was rendered stone-free on the right side.

**Figure 2:**
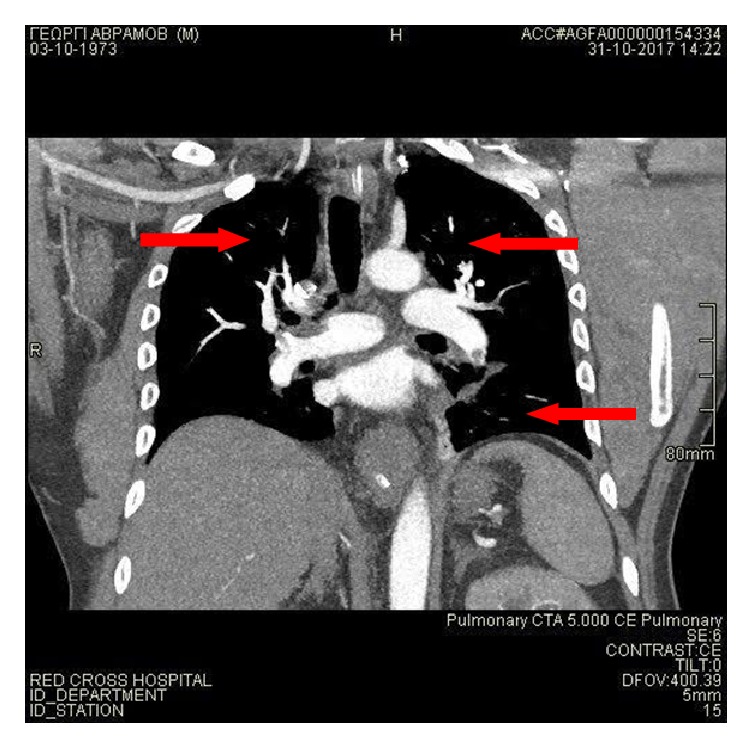
Chest CT angiography scan, depicting filling defects in subsegmental branches of the right pulmonary lobes, in segmental branch of the left lower pulmonary lobe, and in subsegmental branches of the left upper pulmonary lobe.

On the first postoperative day, the patient developed hemodynamic instability, acute respiratory distress, hypoxia, and loss of consciousness. His vital signs included blood pressure (BP) of 88/48 mmHg, heart rate of 110-120 bpm, and oxygen saturation of 50%, on room air. He was urgently intubated and placed on mechanical ventilation. Clinical findings set the suspicion of PE with shock. Chest CT scan demonstrated filling defects in subsegmental branches of the right pulmonary lobes, in segmental branch of the left lower pulmonary lobe, and in subsegmental branches of the left upper pulmonary lobe (Figure 2). Brain and abdominal CT scans were normal. These findings were indicative of high-risk PE with shock.

The patient underwent immediate thrombolysis, with recombinant tissue plasminogen activator (rt-PA) administration (100 mg Alteplase over 2 hours) in the cardiac care unit (CCU), and became hemodynamically stable. Subcutaneous enoxaparin sodium 12.000 iu twice daily (according to his body weight) and vasopressors were also administered. Subsequently, bedside echocardiography revealed both ventricles with normal size and function and normal values of pulmonary artery pressure. Hematuria through nephrostomy tube was present the first three hours after thrombolysis. Seven hours later, he developed hemodynamic instability and fever (38.6°C). Blood and urine cultures were obtained and Piperacillin/Tazobactam and Amikacin were administered empirically. Inotropic agents and blood transfusion were administered, and the patient was transferred to the intensive care unit (ICU).

During the first hours in the ICU, lower limbs venous ultrasonography was negative for deep vein thrombosis (DVT). On the 3rd postoperative day, an abdominal CT scan was performed due to continuous macroscopic hematuria through nephrostomy tube and a decrease in hemoglobin levels, despite blood transfusion. CT scan demonstrated a sizable right retroperitoneal hematoma and the patient underwent angiography and angioembolization of the lower segmental right renal artery. Moreover, the postoperative course was complicated by recurrent respiratory and urinary tract infections due to Acinetobacter Baumanii, Pseudomonas Aeruginosa, Klebsiella pneumoniae, and Klebsiella Oxytoca, which were isolated on sputum, blood, and urine cultures. Antibiotics were administered according to the sensitivity analysis.

The patient developed progressive recovery and was transferred back to the urology department on the 66th postoperative day and was discharged on the 73rd postoperative day. Apixaban 5 mg, twice/day, was orally administered for six months, following the instructions of cardiologists.

## 3. Discussion

PCNL is a well-established treatment option for patients with large, multiple, or inferior calyx renal stones [3–6]. Parameters that influence the complication rate are the surgeon's experience, the operative time, the stone's size and opacity, the number of punctures or tracts, and the presence of bacteria within the stone [3, 4, 6]. PE after PCNL is an uncommon complication. This is the first incident in our department since 2006, in our series exceeding 400 cases. Tae Seung Shin et al. reported one death from PE in their series of 698 patients who underwent PCNL [7], while Hentschel et al. reported one death in their series of 158 cases [8]. S.V Krishna Reddy et al. 2016 report that overall mortality of PCNL due to urosepsis, hemorrhage, or PE ranges from 0,5 to 1,1% in a series of 367 patients that underwent PCNL from a single surgeon [9]. According to the literature, rare cases of air embolism and stone fragment migration through venous system, causing pulmonary embolism, are reported [6, 10].

Acute pulmonary embolism represents the sudden obstruction of the pulmonary arterial vasculature which is usually caused by embolization of thrombus from the deep veins within the lower limbs and pelvis [11]. Although no predisposing factors are identified in approximately 20% of patients (idiopathic or unprovoked PE), most patients have either patient-related or setting-related attributable risk factors (secondary or provoked PE). Patient-related factors include advanced age, previous venous thromboembolism, active cancer, underlying coagulopathy (including factor V Leiden and prothrombin mutations), smoking, hormone replacement therapy, and oral contraceptive pill. Medical conditions associated with an increased risk of PE include heart failure, sepsis, stroke, respiratory failure, and inflammatory bowel disease. Setting-related risk factors include protracted immobility secondary to major general or orthopedic surgery, major fracture, air travel, pregnancy, chemotherapy, or the presence of a central venous line. Commonly, more than one risk factors are present [11], unlike our case, in which no risk factors were present.

PE may be classified as high risk, intermediate, or low risk. When patients with acute pulmonary embolism present with hypotension defined as a systolic blood pressure <90mmHg or drop of >40mmHg, not explained by another cause, this is referred to as high risk PE in the European Guidelines [12, 13]. Thrombolytic therapy should be administered unless contraindicated, as the 30-day mortality risk is greater than 15% in this population. Recent surgery comprises an absolute contraindication to thrombolysis. However, absolute contraindications to thrombolysis might become relative in a patient with immediately life-threatening high-risk PE [13]. American College of Chest Physicians guidelines suggest that recent surgery, excluding recent brain or spinal surgery or trauma, is a relative contraindication and that the bleeding risk reduces significantly 2weeks after surgery. [12].

International literature reveals a paucity of data concerning the experience of thrombolysis following urologic surgical procedures. Cincin et al. report a case of high-risk PE treatment, through ultrasound assisted transcatheter thrombolysis using rt-PA and low-dose unfractionated heparin (LDUH), in a patient with recent radical prostatectomy: a suggested treatment in intermediate and high-risk patients, with high chance of bleeding due to recent urologic surgery [14]. A review of 126,891 cases with venous thromboembolic events (VTE) following urological procedures showed an incidence of VTE to 0,66% (PE 0,37%). For PCNL especially the incidence of VTE is 0,55%. Procedures in supine position seem to have higher rate of VTE compared to lithotomy position. Prolongation of thromboprophylaxis in high risk patients and deprivation of pharmaceutical thromboprophylaxis in low-risk procedures are suggested [15].

A review on VTE in urologic surgery indicates that low molecular weight heparin (LMWH) treatment for postoperative PE is preferred due to rapid approach of therapeutic levels, decreased recurrent bleeding, thrombosis, and mortality and more effective thrombus size reduction compared to LDUH, while LDUH is preferred in postoperative patients undergoing PE with shock and high risk of bleeding, and in cases of renal impairment. The duration of anticoagulant treatment in a first episode of postoperative PE with no identifiable risk factors is 6 to 12 months [16].

Patients undergoing stone surgery should be stratified into groups according to the risk of bleeding and VTE [17]. Thromboprophylaxis with subcutaneous LMWH until complete mobilization is recommended in high-risk patients performing lithotripsy procedures [18].

Although it is a rare complication after percutaneous nephrolithotomy, it requires high index of suspicion so as not to be missed as a diagnosis, especially in hemodynamically unstable patients with respiratory distress. Early diagnosis followed by proper therapeutic actions can be crucial for patients.
